# Recent Advances in Biodegradable Polymers and Their Biological Applications: A Brief Review

**DOI:** 10.3390/polym14224924

**Published:** 2022-11-15

**Authors:** Saleh O. Alaswad, Amira S. Mahmoud, Prabhakarn Arunachalam

**Affiliations:** 1 Nuclear Science Research Institute (NSRI), National Center for Nuclear Technology, King Abdulaziz City for Science and Technology (KACST), Riyadh 11442, Saudi Arabia; 2 Department of Environmental Studies, Institute of Graduate Studies and Research, Alexandria University, Alexandria 21526, Egypt; 3 Chemistry Department, College of Science, King Saud University, Riyadh 11451, Saudi Arabia

**Keywords:** biopolymer, biodegradability, tissue engineering, drug delivery

## Abstract

The rising significance of the field of biopolymers has driven the rapid progress of this distinctive class of polymeric materials in the past decades. Biodegradable polymers have acquired much attention because they play an essential role in humans’ lives due to their specific tunable electrical conductivity and biodegradability characteristics, making them fascinating in many applications. Herein, we debated the recent progress in developing biodegradable polymers and their applications. Initially, we introduce the basics of conducting and biodegradable polymers, trailed by debates about the effective strategies currently used to develop biopolymers. Special importance will focus on the uses of biodegradable polymers in drug delivery and tissue engineering, as well as wound healing, demonstrating the recent findings, and uses of several biodegradable polymers in modern biological uses. In this review, we have provided comprehensive viewpoints on the latest progress of the challenges and future prospects involving biodegradable polymers’ advancement and commercial applications.

## 1. Introduction

Nowadays, plastics are a vital part of human society in our daily lives and are widely used in countless industries, such as packaging, building materials, textiles, transportation, healthcare, and so on [1,2]. Furthermore, plastics are considered moisture-resistant, bendable, durable, and, specifically, more economical than any other material. Particularly, utilization and manufacturing have considerably upsurged for nearly half a century due to their features. After that, 8.3 billion metric tons of plastic materials have been manufactured globally, with plastic materials occupying a ubiquitous role in our daily lives [3]. Notably, worldwide plastic production almost reached 370 million tons in 2019 and it is anticipated to substantially evolve over the coming decade [4]. More importantly, plastic material has considerably impacted the areas of medicine, space programs, transportation, and life-saving devices such as incubators, helmets, ventilators, and carriers for safe drinking water [5]. Excessive quantities of plastic waste are produced in both developing and developed countries. Furthermore, plastic materials’ resilience and non-biodegradable features have allowed them to remain in the ecosystem over a more extended period, instigating the most stable waste product over other methods of waste. However, the continual need, mishandling, and littering of plastics became destructive. More importantly, petrochemically derived plastics damage the environment even after disposal. Polyethylene, polypropylene, polyvinyl chloride (PVC), polystyrene, polycarbonate, and polymethyl methacrylate can be cast under a thermal process and labeled as plastics [6]. Notably, plastic materials tend to contain harmful chemicals, so land and water accretion instigate severe ecological damage. Generally, plastics are derived from petrochemical origins, contributing to greenhouse releases. As plastic materials are developed for flexibility, durability, and non-degradability, they have damagingly affected every part of the ecological system, killing plant life and posturing risks to local animals and human communities.

Nowadays, ecological alertness and environmental impact related to fossil-based plastics assets have led to more studies and research into developing bio-derived plastic materials. The term is labeled by European Bioplastic materials as either biodegradable plastics [7,8], and/or plastics created from renewable sources. Currently, bioplastics account for almost 1% of the total plastics manufactured yearly, but according to estimates about future progress, the total production capacity of six million tons will be reached in the coming years [9]. Biodegradable polymers are generally derivatives of renewable resources and are considered abundant. Biopolymers have several benefits over conventional plastics due to their non-toxic nature, biocompatibility, degradability, sustainability, and extreme hydrophilicity [10]. The chief growth drivers in biopolymers are polyhydroxyalkanoates (PHAs) and polylactide (PLA) [11].

Biodegradable polymers can be applied in the medicinal arena and are mainly classified into drug delivery systems [12,13], wound healing products [14,15,16], and surgical implant devices [17]. The advancement of biopolymeric drug delivery systems now attains remarkable interest, especially in controlled delivery. More importantly, drug delivery inside humans can be regulated through biodegradable capsules [18]. Particularly, biodegradable polymers are used to prepare novel formulations, and the high permeability of buccal mucosa is an appropriate target for drug delivery [19]. In this regard, drug delivery combined with biopolymers and buccal routes is shielding, safe, and rapidly functioning. Similarly, in the case of wound healing, bioresorbable non-wovens to substitute human tissue repair [20], and simple sutures, staples, or meshes, are accessible [21,22]. Comparatively, the usage of biological resorbable scaffolds for tissue engineering [23] is worth revealing. Further, biodegradable polymers are renewable, cost-effective, and found in various varieties [24]. Notably, biodegradable polymers are considered an exceptional candidate for wound healing due to their bioactive features, facilitating cell growth and regeneration potential, and providing antimicrobial conditions and immunomodulation [25]. In addition, biodegradable polymers are a probable candidate for wound care because they can absorb a massive amount of water. In recent years, these polymers are capable of releasing drugs at the site of damage and making them appropriate for healing applications. Many biopolymers have good film-forming features, making them applicable for conventional commodity applications [26]. Notably, they are used in foodstuff containers (bottles, jars), soil retention sheeting, farming film, garbage bags, and wrapping material [27]. Additionally, biodegradable polymers in non-woven form can be used in farming, filtration, hygiene, and protecting wear [28].

This discovery was the turning point in the upsurge of research works into biodegradable polymer; although it has not been completely utilized in different applications. Particularly, biodegradable polymers are mainly used in drug delivery applications and are very stimulating because of compatibility concerns of biopolymers with tissue and release kinetics of these polymer particles. More importantly, the drug release is determined by the depletion features of the polymer’s drug release. Furthermore, these biopolymers break down and are removed from the human body after they have performed their projected work. Mainly, biopolymers must be destroyed at a rate comparable to the healing and regeneration process. The biodegradable polymer features have been shown to possess desirable and tenable mechanical features [29]. The biodegradable polymers must possess biocompatibility features and must not elicit immune reactions. Moreover, the degraded products must be non-toxic, metabolized, and eradicated simply. The fabricated polymers must be easy to synthesize and keep significant durability to withstand the fabrication process [30,31].

**Figure 1:**
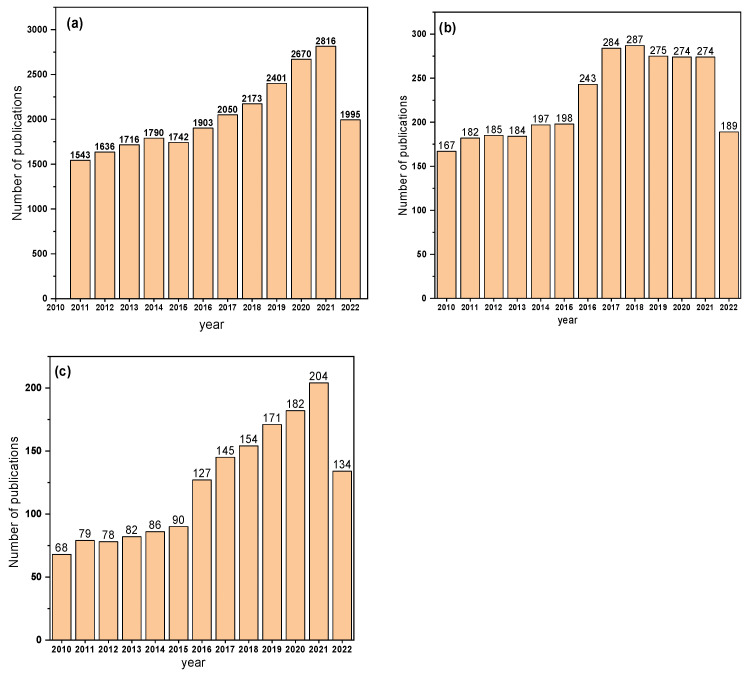
The number of studies from 2010 to 20 October 2022 on (**a**) biodegradable polymers, (**b**) biodegradable polymers and drug delivery, and (**c**) biodegradable polymers and biological applications from the web of science database.

In the meantime, significant research works have been dedicated to developing biodegradable polymers such as PHA, PLA, starch, cellulose pulp, and others. In past decades, these biodegradable materials have been examined for biological applications, and the number of publications counts is nearly equal to or higher than what existed before. As for biodegradable polymers, PHAs are applied in households, farming, industrialized, and medicinal arenas [32] due to their physicochemical features of water insolubility, UV light resistance, and a good barrier from oxygen penetration [33]. Furthermore, Figure 1 shows the number of research works in biodegradable polymers from 2010–2022, based on the Web of Science search using the keyword “biodegradable polymers”.

This list of promising applications of these biopolymers is by no means comprehensive. Indeed, the quantity of potential uses is almost infinite. It also has to be stated that most of the uses involve the biodegradable nature of biopolymers. Biopolymers along with their significance in different applications are summarized in Table 1. Biological applications have been selected as a focus area for biodegradable polymer use because they retain their greater entity impacts. Biodegradable polymers are being gradually applied to medical applications, particularly in medical implant applications. More importantly, biopolymers can be used as surgical sutures, offering admirable durability and sturdiness; they can be applied in tissue regeneration applications. Additionally, the biopolymers suture materials can be easily removed or permitted to readily disappear from the body. For instance, Poly-(glycolic acid) (PGA), poly-(L-lactic acid) (PLA), and their composite materials are widely applied as sutures as they provide reliant knot steadiness and remarkable flexibility [34,35].

We have tried to provide a full span of knowledge in a lone review, contrary to the numerous earlier reports which treat the topics individually. In this review, we examine the different kinds of applications of biodegradable biopolymers. Likewise, we will provide an overview of all the potential applications, looking at their availability, cost, biodegradability, easiness, and affinity to specific features of biopolymers. This report provides a brief summary of biopolymers and their features, followed by the latest developments in their biological applications, with a focus on drug delivery systems, wound healing, and prospects brought about by the design and application of biodegradable polymers in biological applications. Finally, this review is timely to debate the different applications of biopolymers and critically observe the particular properties of numerous biopolymers.

## 2. Categorization of Biodegradable Polymers

**Figure 2:**
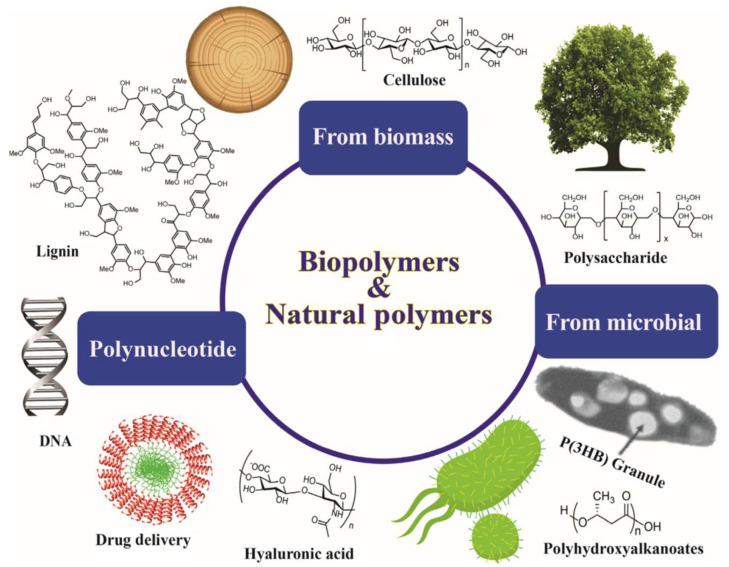
Chemical structures of the selective biopolymers and their classifications [56].

The biopolymers are degraded to low-molecular-weight compounds under the action of micro- and/or microorganisms or enzymes. Biopolymers such as PCL and PLA, amide-rich biopolymers, polyurethanes (PU), and most natural biopolymers have heteroatoms, which are possibly liable to hydrolytic cleavage of the ester group, and amide groups [51]. Based on the source of raw ingredients (obtained through natural sources, viz, sugar, starch, cellulose, and fossil oil), biodegradable polymers can be classified into three sets, namely (1) natural; (2) synthetic; and (3) microorganism built biodegradable polymers [52]. Natural biopolymers are made from natural resources, whereas synthetic polymers are from petroleum sources. Mostly, we stated the most commonly explored aliphatic polyesters and usual natural macromolecules. In particular, the chemical structures of particular biopolymers are presented in Figure 2. Typically, the chemical structure of natural macromolecules and their major source are shown in Figure 2. For instance, lignin and polysaccharides are the most extensively explored natural macromolecules. The case of polysaccharides mostly involves cellulose, chitin, lignin, chitosan, sodium alginate, and so on [53].

We consider that with continual research efforts, each natural macromolecule will be significantly advanced, involving sources, extraction, and filtration setup. They will be obtained through their adaptation and product development;, and natural macromolecules will certainly assist humans in various fields. Further, other types of biodegradable polymers, such as biodegradable PU, amide-rich polymers, such as polypeptide and thermal polyaspartate and so on [54,55]. Generally, polypeptides, DNA, and protein are the research areas in the arena of life science, and they are hardly applied in the environmental aspects (Figure 2).

## 3. Promising Areas of Biopolymer Applications

The usage of biopolymers is rapidly emerging with a worldwide economy worth many billions of dollars yearly. A varied series of areas where uses for biodegradable polymers have been applied involve medicinal, packaging, farming, and the automotive industry [57]. In addition, biodegradable polymers that can be applied in packaging continue to gain more consideration than those used for other applications. It is projected that 41% of plastics are used in packaging and that nearly half of that volume applies to wrap food materials [58]. The reusable and degradable features of biopolymers are what make them tempting for inventive use in packaging. In particular, biopolymers are generally used in different industrialized applications, such as food wrapping, cosmetics, and medicine [59]. Biodegradable polymers have a lower solubility in water and a very imperative water uptake, thereby they can be applied as absorbent candidates in biological, healthcare, horticulture, and farming applications. Particularly, biodegradable polymers have been used in certain applications in which plastics cannot be used, such as making artificial tissue. These uses might demand biomaterial features such as biocompatibility, environmental responsiveness, and biodegradable candidates with sensitivity to variations in pH and physicochemical and thermal variations [60]. Generally, biopolymers display poor thermal and mechanical features (tensile strength and brittleness), chemical resistance, and processability than synthetic polymers. Particularly, to make them appropriate for specific uses, they can be reinforced with fillers that drastically enhance these features.

Biodegradable polymers play valuable roles in several human body functions, such as embracing cells to create tissues and signaling the cells to regulate their features. They also moderate the skin’s hydration and elasticity to keep its natural state. Additionally, they enable all joints and gastrointestinal tracts to be flexible through lubrication and protection from pathogens by accumulating into the mucus gel covering the human eyes and respiratory tract [61]. This section debates the favorable areas for using biodegradable polymers and their composite materials with setbacks, state-of-the-art methods, and innovation in carriers.

## 4. Drug Delivery

In recent years, drug delivery research and its advancement have been considered to be of vital significance in biomedical and healthcare uses [62,63,64,65]. Nowadays, developing competent drug delivery systems is a major concern for drug delivery researchers. Drug delivery is a method of directing a pharmacologically active compound to achieve a therapeutic effect in humans. Notably, various drug delivery methods have been advanced and explored at the target site for drug delivery [66]. For handling human-related illnesses, many paths include drug targeting systems for oral, nasal, ocular, and transdermal routes [67]. Biopolymers have developed certain applications in drug delivery in the form of beads, solid monolithic matrix systems, films, implants, micro and nanostructured particles as well as inhalations and injectable systems, and viscous liquid formulations. Different anatomical routes are recognized as safe and reliable for administering numerous medical drugs to the human body. The selection of the drug delivery paths depends on the following disputes, (a) the effect anticipated by the kind of the disease; (b) the kind of the product; (c) the drug will be openly directed to the organ that hurts from the disease [68]. In past decades, targeting drug delivery has amassed attention, and many approaches were established to boost the targeting of drug delivery by developing numerous nanoparticle-based systems.

The invention in biomaterial fields and engineering permits the examination and utilization of numerous developed biomaterials, keeping their essential biomaterial features like biodegradability, biological compatibility, environmental alertness, etc. Biomaterials are changed or subjected to functionalization to develop these promoted candidates for enhanced drug delivery. Biodegradable polymers exist as beads, films, microparticles, and solid monolithic matrix systems; nanoparticles are applied and used via different routes targeting specific sites/cell populations in organs like the lung, kidney, and pancreas for the drug release in an established fashion, and deterioration in a limited period.

**Figure 3:**
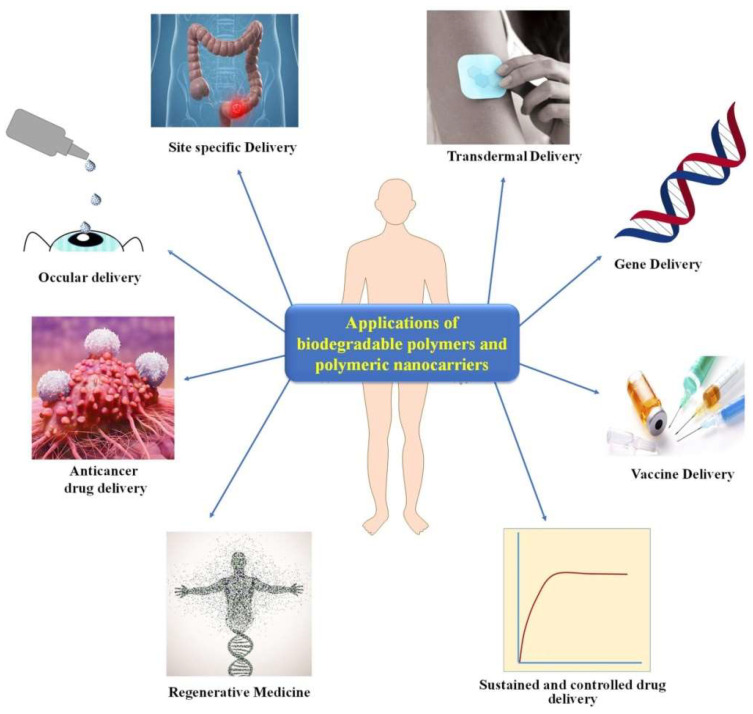
Different kinds of biopolymer applications and polymeric nanocarriers [74].

In general, the greatest advantage of biopolymers is their ability to degrade under aerobic and anaerobic conditions. In particular, biopolymers in nanocarriers have fascinating considerations because they degrade within the body in physical circumstances. In addition, biopolymers can be considered for medical uses; specifically, drug-related uses. Protein-related biopolymers have been introduced as essential devices (Figure 3) [69]. Many polysaccharides, namely chitosan, chondroitin sulfate, cyclodextrin dextrin, insulin, amylase, and locust bean gum have been considered for drug delivery applications [70,71,72,73].

In general, biopolymer’s drug delivery applications include oral, nasal, and parenteral; in addition, transdermal management of drug delivery, protein and gene delivery, and the usage of implants to are used to treat illnesses [74,75,76,77]. Several aliphatic polyesters and cross-linked polyorthoesters have been explored in drug delivery use, especially in antineoplastic agents, antidiabetics, vaccine delivery, etc. More importantly, synthetic peptides are enzymatic decomposable peptides that can create cross-links in synthetic hydrogels [78]. Similarly, new phosphazenes-based polyphosphoester must have a characteristic backbone comprising P-atoms associated with carbon/oxygen. Particularly, phosphazine biopolymers and copolymers can encapsulate hydrophilic and lipophilic drugs [51]. Moreover, these polymers can potentially deliver the targeted site of antineoplastic agents when folic acid is introduced as a targeting ligand to the polymer [79]. Further, this aliphatic polyester is a hydrophobic material, and PLA is demonstrated here. More importantly, PLA uses in drug delivery are mainly categorized by synthesizing new kinds of block copolymers as biocompatible hydrophobic fragments and preparing micelles or polymer microspheres for drug incorporation. In particular, Liu et al. demonstrated that the PLA—PCL holding block copolymer and the obtained micelles have good durability, remarkable drug filling, and a pH-selective governable release feature [80]. Further, it also tends to have combined drugs (e.g., benzoxaborole drugs) in polymeric materials in the PLA matrix, and novel kinds of antimicrobial and antiparasitic drugs [81]. PCL surgical sutures and loaded other medicines to attain hemostasis, bacteriostatic, and added effects are examples of characteristic drug-integrated medical uses.

Chitosan-based biodegradable polymers show good absorption, organized release, and bioadhesive features [82]. Furthermore, these biopolymers possess mucoadhesivity, biodegradability, biocompatibility, and have remarkable biological features that make chitosan appropriate for drug delivery methods with greater biodistribution, selectivity, and minimal harmfulness [83]. The deacetylation degree is in the range of polymer-side chains that can be manipulated for target-precise drug delivery uses [84]. The polymer solution vaporizes to produce polymer layers, and these layers work as a protecting coating for tablets holding a sensitive drug for release [85]. The primary concern for advancing scientific outcomes is the target-specific delivery of therapeutic drugs to the specific diseased tissue by nanomaterials when enhanced permeability and retention (EPR) is not appropriate. To overcome this, nanomaterials are surface-tuned through a ligand as a directing moiety; this ligand reveals distinctive targeting capabilities to direct selective binding of nanomaterials and reduces non-productive delivery. The essential potential of controlled drug delivery through biopolymeric nanomaterials is to advance efficacy by escalating drug concentrations at the targeting site while simultaneously curtailing harmfulness by reducing target accumulation. The current development of nanomaterials to enhance passive targets is mostly based on the improved ability of active molecular targeting. In targeted drug delivery, enriched receptor-mediated uptake is attained by engineered nanomaterials’ surfaces with ligands [86,87].

Biopolymers have been examined as vectors (polymeric) to provide the drug or genetic cargo. Exceptionally, different biocompatible natural polymers (chitosan, dextran, hydroxyapatite) can transfect the cell lacking amendment of active directing, permitting it to be cell- or tissue-specific. Compared to hydroxyapatite, dextran sulphate can gather in the liver by binding to receptors that exist on sinusoidal endothelial cells [88]. Covering chitosan over Poly(lactic-co-glycolic acid) (PLGA) materials enhances the antiepileptic effectiveness through intranasal delivery since the chitosan materials can bind with ciliary, enhancing nanomaterials retaining over the surface [89].

The above-mentioned biodegradable polymers have been extensively investigated for numerous drug delivery uses by means of in vitro/in vivo examination. Despite significant research on different existing routes of drug delivery, still, more efforts are needed to examine the possibility of buccal-mediated biopolymeric delivery of the drug. We expect that further examination of explored biodegradable polymers as targeted drug delivery agents will open a vista of using these biopolymeric materials for the advancement in biological applications.

## 5. Wound Healing Applications

**Figure 4:**
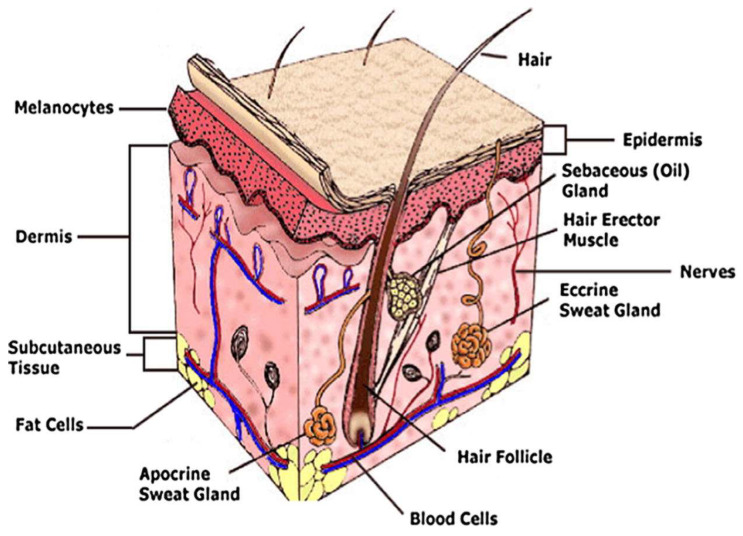
Graphic illustration of typical skin structure, involving three layers: epidermis, dermis, and hypodermis [96].

The wound healing method is complex, involving a cascade of biological processes instigated with respect to an injury. In particular, these biological reactions involve the interaction of immune and non-immune cells, soluble mediators, and extracellular constituents [90,91]. The degree of the healing process mainly depends on the patient’s immunological status. Usually, wounds under natural conditions heal independently by four overlapping stages; hemostasis, inflammation, proliferation, and remodeling [92]. Depending on the depth and degree of the skin injury, it heals by major closure, secondary wound healing, or delayed primary closure [93]. More importantly, the vertebrate body’s major organ is the skin, providing an external defense system alongside exterior attacks such as infection and the effect of severe outward situations. It is also crucial to protect its part in sensory detection, controlling body heat, fluid homeostasis, and self-healing. Generally, skin comprises three segments. Thin and higher cellular epidermis, which functions as a fence to the body and regulates the fluid loss in the wounds. Subsequently, the acellular dermis, which typically comprises the collagen-rich extracellular matrix, provides physiological strength and flexibility for the skin; it lodges the nerve bundles, vasculature, and lymphatic system [94,95]. Furthermore, the hypodermic fat tissue’s inner layer, termed the hypodermis, offers thermal isolation and mechanical defense from external wounds, as shown in Figure 4.

Biodegradable polymeric materials are recognized as appropriate candidates for wound healing applications. It is well known that biopolymers have a wide-ranging usage in wound dressing relating to injury owing to their biocompatibility features and biodegradable nature, and their similarity to extracellular matrices [97,98]. The major origin of these natural healing candidates is living things such as fungi (chitin), algae (alginate), bacteria, plants, and animals (chitosan, collagen) [99]. A sequence of reiterating parts held by diverse covalent bonds forms these biodegradable polymers, namely monomers of amino acids, nucleotides, monosaccharides, etc. Further, these biodegradable polymers can quickly be saturated with biological fluids concerning their three-dimensional network structure. These ingrained curing features make them an appropriate material for pharmaceutical and medicinal uses, specifically for wound healing applications, tissue engineering using drug delivery, and implants [100]. Different kinds of chitosan-based material dressings also exist globally. Dextatic and dextrosan have hemostatic and antibiotic features. Opticell is applied for partial and thicker wounds, first-st and second-grade burns, diabetic foot ulcers, pressure ulcers, medical wounds, donor sites, and arterial and leg ulcers. Additionally, other kinds of biodegradable polymers, namely starch, glucan, dextran, and silk, have reported their applications in wound healing.

However, these improved methods are reasonably effective and need to be regularized for patients with chronic wounds for advanced and efficient wound healing. Generally, we anticipate that an improved classification approach for different wounds, the improvement of novel and developed approaches for finding cellular alteration and diversity, as well as other technical developments, will benefit in attaining promising, active, and clinically substantial wound healing treatments. The full potential of these materials and their ability to aid wound healing need more comprehensive examination.

## 6. Medical and Hygiene

Biodegradable polymeric materials are considered for medicinal purposes for suturing, covering, fixation, isolation, organized drug delivery, contact inhibition, and tissue guidance. PLA, poly-(glyconic acid), and copolymers are generally applied in suturing owing to their exceptional flexibility and reliance on knot strength [101]. Poly(orthoester) and poly-hydroxy sets are mainly involved in drug delivery treatments. Further, PU materials have flexibility and more substantial wear and tear characteristics, which are highly required for grafting scaffolds assisting synthetic blood vessels [102]. Notably, PEA materials have decent thermo-mechanical features and enable drug delivery, hydrogel, and tissue engineering uses. More importantly, hospitals mostly engage in hygiene and health-related materials like surgical masks/gowns, gloves, sterile napkins, diapers, bedding, medical scrubs, nursing uniforms, antimicrobial textiles, wipes, etc. Biobased PET can be used in surgical gowns in place of conservative cotton, polyester, and PE [103]. In addition, PLA makes gowns, caps, and masks [104]. Thermoplastic starch is applied for creating disposable diapers owing to their super absorbent features [105,106]. Furthermore, biodegradable materials, including alginate fibers, catgut, collagen, chitosan, and super absorbent polymer, are applied mostly for medical and hygiene uses. Table 2 illustrates the different kinds of uses of biodegradable polymers in the medicinal and hygiene arenas. It is expected that these numerous regenerative medicine strategies will translate from ‘bench to bedside’ in the future. Although, significant research works are required to improve the mechanical features of biomaterials that are essential for this type of biological application. Notably, the ability of biodegradable polymers has been examined by several researchers for various kinds of biomedical uses credited to their physiochemical features, such as biocompatibility and biological safety. Lastly, it might be anticipated that humans will remarkably benefit from nanotechnology-based nanomedicines in the coming years for the management and treatment of fatal diseases, particularly in cancer therapy.

## 7. Future Improvements

Substantial research works have been dedicated to the development of biopolymers over the past decades. Mainly, the advancement of biodegradable polymers and their composite has been gaining amazing limelight, however, it is still in earlier phases. The requirement for new kinds of candidates from future manufacturers of biodegradable polymers is irresistible. In particular, while there occurs a grave requirement for a colossal array of sustainable products, poor performance features, and high development costs drop their production in relation to classical synthetic polymers. Renewed ecological norms focused on resolving eco-centric trepidations have caused improvements toward the development of modern polymeric materials and processes that are agreed to obey the welfare of normal habitats. However, it is necessary to develop highly active biodegradable polymer products and exploit the ecological, social, and industrial benefits.

One of the key setbacks of biopolymers obtained from renewable resources is their rapid rate of degradation owing to their hydrophilic nature and, in definite conditions, low mechanical features, particularly in water environments. However, obtained biopolymers have several benefits since they are acquired from a wide range of plant components. Notably, in recent years, bio-derived polyesters have been receiving much consideration based on biodegradability as well as probable medical applications. Explicit features such as biodegradation methodologies, biocompatibility, operating conditions, and potential usages in medicine, ecological protection, and agro-chemistry have expected a lot of attention. The biological safety of developed biopolymers and the nano-safety of their composite continue to be insignificant. This novel perception emphasizes expecting, assessing, and demonstrating possible concerns related to advanced polymer usage. In the future, major setbacks of biodegradable polymers must be associated with managing primary materials, the performance of bio-derived materials, and their production cost. Furthermore, commercial manufacturing will be another challenge for the manufacturing of bio-derived monomers and bio-derived polymers from renewable sources. Constructing industrial plants can be demanding due to the lack of experience in new technologies and the assessment of stock/demand balance. Although new kinds of bio-derived polymers are developed on an industrialized scale, there are still various issues that need to be determined for the long-standing feasibility of biodegradable polymers. It is anticipated that there will be feedstock competition as a global requirement for food and energy to upsurge over time.

## 8. Conclusions

Herein, we provided a comprehensive overview of the recent advances in the biological applications of biodegradable polymers. Eco-friendly biopolymers are favorable candidates for the development of emergent applications with the merits of biodegradability and biocompatibility. Still, biopolymers reveal supremacy as they can be eroded into small trashes and can effortlessly be expelled out of the human body. In recent years, significant biodegradable polymeric drug delivery systems have been realized in biological applications [107,108]. However, more detailed research efforts must be performed for the development to choose appropriate drug delivery methods with greater reliability. However, the in-depth understanding of the mechanistic features and time taken for the drug delivery system for a tissue related to their objective is acute. These are the setbacks in addressing the uncertain medical need over time. In the future, implementing different kinds of biodegradable polymeric methods in therapeutic uses, including scaling up with organized and targeting activities, might be a considerable stage as the appropriate nanocarrier materials are mandatory to transport the genetic material to the target-specific region. In this regard, a highly active synthetic approach for developing such biodegradable polymeric systems must be highlighted as they will be applied more in biological methods. All the available methods arise with demerits when applied to explore biodegradable polymers. To conclude, this is a timely evaluation to debate biodegradable polymers and critically explore their significance and their applications.

## Figures and Tables

**Table 1:** Various kinds of biopolymers and their biological uses.

Biopolymers	Materials	Area of Applications	Ref.
PLGA (Poly(lactic-co-glycolic acid))	Polyethylene glycol/PLGA	Advanced cellular uptake and hypoglycemic effect on oral administration.	[36]
	Polysorbate-80-coated PLGA	Reduced hemolysis, improved antioxidant activity	[37]
	paclitaxel loaded PLGA nanoparticles	Enhanced internalization, and targeting potential	[38]
	Polysorbate-80-coated PLGA	Enhanced mechanism of drug delivery, diminished drug dosage, and side effects	[39]
PLA (Polylactic acid)	Docetaxel loaded PEG-PLA	Enhanced cytotoxicity and apoptosis	[40]
	Docetaxel loaded PLA	Momentous decrease in tumor size and cell proliferation	[41]
	Tamoxifen loaded PLA	Noteworthy decrease in renal toxicity, hepatoxicity, and immunogenic side effects	[42]
	Glimepiride loaded PLA	Improved continuous drug release effect	[43]
Albumin	Folate-conjugated albumin	Enhanced target-site potential to the activated macrophages cells	[44]
	Albumin NPs decorated newly selected anti-Met nanobodies	Enhanced lysosomal drugs delivery into Met-positive tumor cells	[45]
Polycaprolactones (PCL)	hydroxyl telechelic natural rubber/poly(ε-caprolactone) diol	Drug delivery systems	[46]
	Biodegradable PCL	Ethicon: monocryl-suture, Capronor-contraceptive implant	[47]
Polybutyrate adipate terephthalate (PBAT)	Polymer blends of PLA/PBAT	Packaging applications Bottle uses	[48]
Polyesteramides (PEA)	PEA	Hydrogels Drug delivery Tissue engineering Smart materials (T sensitive)	[47]
Polyhydroxyvalerate (PHV)	PHV	Obs: biodegradability 3–12 months Dung bags, customer packing materials, agriculture film, Rubbermaid, Calphalon, PaperMate, BioTuf	[47]
Poly(alkylenealkanoate)s (PBS)	PBS	Injection molding, one-use goods (spoons, forks), fibers, fishing gear, plant pot.	[49]
Thermoplastic starch (TPS)	TPS	Package applications	[50]

**Table 2:** Probable biodegradable polymer applications in the medical arena.

Medical Uses	Materials Applied	Probable Biopolymer Replacement
Suture	Polyamide, polypropylene, Poly(vinylidene fluoride)	PLA, PGA, PLGA
Wound bandage	Polyvinyl alcohol (PVA), cotton	PGA, PLA
Blood bags	PVC	PBAT, PHB
Catheters	High-density polyethylene, Poly (dimethylsiloxane), polyether ether ketone, PP	PLA, PGA, PLGA, TPS, PCL
Plasters	Toluene 3, 4 diisocyanate and polyethylene glycols (Lycra fibers)	PLA, TPS
Surgical gowns Cotton, polyesters	PP, PE	Biobased (PET, PBT)
Caps, gowns, surgical masks	PET, cotton	PLA, TPS
Clinical hosiery	PET, cotton, PP, PE	Biobased PET
Pillow cover	Polyesters	PLA, TPS
Medical scrubs	Polyesters	Biobased PET, TPS, PLA
Disposable diapers	Polyacrylic acid, PVA copolymers	TPS

## Data Availability

Not applicable.
